# Identifying Changes in the Synaptic Proteome of Cirrhotic Alcoholic Superior Frontal Gyrus

**DOI:** 10.2174/157015911795017164

**Published:** 2011-03

**Authors:** N Etheridge, R.D Mayfield, R.A Harris, P.R Dodd

**Affiliations:** a School of Chemistry and Molecular Biosciences, University of Queensland, Australia; b Waggoner Centre for Alcohol and Addiction Research, University of Texas, USA

**Keywords:** Alcoholism, cirrhosis, proteomics, post-translational modification.

## Abstract

Hepatic complications are a common side-effect of alcoholism. Without the detoxification capabilities of the liver, alcohol misuse induces changes in gene and protein expression throughout the body. A global proteomics approach was used to identify these protein changes in the brain. We utilised human autopsy tissue from the superior frontal gyrus (SFG) of six cirrhotic alcoholics, six alcoholics without comorbid disease, and six non-alcoholic non-cirrhotic controls. Synaptic proteins were isolated and used in two-dimensional differential in-gel electrophoresis coupled with mass spectrometry. Many expression differences were confined to one or other alcoholic sub-group. Cirrhotic alcoholics showed 99 differences in protein expression levels from controls, of which half also differed from non-comorbid alcoholics. This may reflect differences in disease severity between the sub-groups of alcoholics, or differences in patterns of harmful drinking. Alternatively, the protein profiles may result from differences between cirrhotic and non-comorbid alcoholics in subjects’ responses to alcohol misuse. Ten proteins were identified in at least two spots on the 2D gel; they were involved in basal energy metabolism, synaptic vesicle recycling, and chaperoning. These post-translationally modified isoforms were differentially regulated in cirrhotic alcoholics, indicating a level of epigenetic control not previously observed in this disorder.

## INTRODUCTION

Excessive consumption of alcohol affects many parts of the body, including the brain and the liver [1]. Liver damage amongst alcoholics ranges from reversible steatosis characterised by impaired metabolic and detoxifying functions, to irreversible, and often fatal, micronodular cirrhosis [2]. Cirrhosis of the liver is not an inevitable outcome for alcoholics: many factors influence the progression from fatty liver to cirrhosis, including gender, genetics, nutrition and level of alcohol intake [2]. In consequence, cirrhosis is not found in all alcoholics; however, alcoholism is the most common cause of cirrhosis in developed countries [3].

The recent explosion in the application of proteomics to the study of neurodegenerative disease can be attributed to rapid technological advances in methodology. The ‘classical’ proteomics approach consists of two-dimensional electrophoresis (2DE) coupled to mass spectrometry (MS), and is still a powerful tool for comparative protein profiling. It is particularly useful in analyses of post-translationally modified proteins, as this ‘visual’ technique enables each protein and its isoforms to be observed on the 2D gel. Post-translationally modified proteins are highly abundant in the human proteome [4]: in some tissues, all gene products are represented by an average of three to five multiple protein expression forms (MPEFs) [5]. Research into the regulation of post-translational modifications is in its infancy, but is beginning to show that regulation of protein level is only a fraction of the changes that occur in the disease state; major changes to MPEF levels in disease are responsible for altered function. The mechanisms regulating these modifications (such as phosphorylation, acetylation, S-nitrosylation, glycosylation, ubiquitin-like-modifier conjugation, etc), which can also be studied with proteomics, are essential parts of the processes by which disease brings about cellular changes.

## MATERIALS AND METHODS

### Case Selection

Subjects were categorized according to alcohol intake. Alcoholics were defined by National Health and Medical Research Council/WHO criteria as individuals who had consumed an average of more than 80 g ethanol/day throughout most of their adult life; the alcohol consumption of controls was < 20 g ethanol/day. Cases with multiple drug use or Wernicke-Korsakoff syndrome were excluded. Controls had no history of brain dysfunction, and no significant brain abnormalities were detected by post-mortem examination. Six controls and six alcoholic cases were chosen and matched as closely as possible for age, sex and post-mortem interval. Subject details are shown in Table **1**.

Samples were collected by qualified pathologists from the Brisbane node of the National Health and Medical Research Council (NHMRC) Brain Bank and the Tissue Resource Centre at the University of Sydney, Australia. Full ethical clearance and informed written consent was obtained from the next of kin.

### Protein Extraction

Synaptosomes were prepared as *per* [6].

### 2D-Differential In-gel Electrophoresis (DIGE)

CyDye labelling and 2-dimensional electrophoresis were performed as *per* [6]. A dye-swapping approach was utilized to minimize the effects of variable CyDye labelling efficiency. In essence, half of each group (control, non-comorbid alcoholics, or cirrhotic alcoholics) was labelled with Cy3, and the other half was labelled with Cy5. An internal standard (IS) was created by mixing 20 µg protein from all subjects, which was divided into 12 × µ16 g aliquots and labelled with Cy2.

### Comparative Analysis

Gels were scanned on a Typhoon 9400 scanner (GE Lifesciences, Princeton, NJ, USA) at three wavelengths to record the fluorescence of each of the three Cy Dyes in the gel. Images were cropped and rotated in Image Quant (BioRad, Hercules, CA, USA), then imported into the TT900 S2S (NonLinear Dynamics, Newcastle upon Tyne, UK) image alignment module for gel/spot alignment of all gels to each other. Aligned gels were imported into Progenesis PG240 (NonLinear Dynamics) then automatically analyzed using the SameSpots module to accurately compare the same protein spot in each gel. Automatic analysis included background subtraction and radiometric normalization. Differences in normalized spot volumes between gels were analyzed in Statistica (Statsoft, Tulsa, OK, USA) using analysis of covariance (ANCOVA) with post-mortem delay and age as covariants. Pairwise differences were assessed with Newman-Keuls *post-hoc* test. Only proteins with expression differences greater than 1.2-fold and Newman-Keuls P values ≤ 0.05 were analyzed in this study.

### Mass Spectrometry

Trypsin digest, matrix assisted laser desorption-ionisation time-of-flight (MALDI-TOF) mass spectrometry and data processing were performed as described in [6].

## RESULTS AND DISCUSSION

The synaptic proteins were isolated from human brain tissue collected at autopsy that had been slowly frozen and stored in isotonic 0.32 M sucrose solution to minimise damage to sub-cellular structures by ice crystals [7]. Both groups of alcoholics had lower mean ages at death (non-comorbid alcoholics: 50.2 ± 5.0 years; cirrhotic alcoholics: 58.7 ± 6.1 years; mean ± SEM) than the controls (68.0 ± 5.6 years); this generally reflects the characteristics of such subjects collected by the Queensland Brain Bank. Although these differences were not significant this partly reflects the spread of values (Table **1**) and the sample size. The groups were quite well matched on the delay between death and autopsy (post-mortem interval; PMI) but the spread in these values was also quite notable (Table **1**). As these factors might influence the amounts of proteins detected on the gels, and also contribute to sample variance, age and PMI were included as covariants in the statistical analysis.

The proteomic analysis aimed to identify two sets of proteins representing the effects on the brain of alcohol misuse combined with liver dysfunction. To do this, we obtained synaptosomal proteins from three groups of subjects. The first comparison identified the set of proteins that differed significantly between cirrhotic alcoholics and normal, non-alcoholic controls. These proteins represent the end result of the effects of chronic alcoholism combined with the additional effects of cirrhosis. Ninety-nine proteins fell into this category. Alcoholics who develop cirrhosis of the liver generally have higher lifetime alcohol consumption than non-cirrhotic alcoholics [2]; hence, the proteins that differ in concentration from controls in frontal cortex may do so simply in response to the severity of the chronic alcohol abuse. However, the set of proteins that are differentially expressed when non-cirrhotic alcoholics are compared with controls are quite different [6], indicating that human brain proteins are regulated differently in alcoholics with comorbid cirrhosis than in alcoholics without liver dysfunction.

This result was further supported when proteins that were differentially expressed between non-cirrhotic and cirrhotic alcoholics were identified. In this second comparison, 118 proteins were differentially regulated; less than 50% of these were the same as those that differed from controls (Fig. (**1**)). Brain proteins in cirrhotic alcoholics appeared to have been regulated in a different manner from those in non-cirrhotic alcoholics. This differential regulation might be due to genetic, nutritional, or other factors; but the data suggest that consumption alone is not sufficient to explain the disparity.

Proteins that showed significant expression differences between cirrhotic alcoholics and the other two groups were identified by their peptide-mass fingerprints (PMF) after trypsin digestion using matrix assisted laser desorption/ionization-time of flight (MALDI-TOF) mass spectrometry. The PMF of each protein was compared against a database containing the theoretical PMFs of all known human proteins. The identification was considered accurate when at least five peptides from the unknown PMF matched a theoretical PMF with a difference in mass of 0.0030% or less. These and other criteria (see Methods) reduced the probability of false identification.

Several of the proteins differentially expressed in cirrhotic alcoholics were identified in more than one spot on the 2D gel (Table **2**). These multiple protein expression forms (MPEFs) are either the result of alternate splicing, or they are post-translationally modified isoforms of the same transcripts [8]. MPEFs represent a large proportion of the human proteome [4, 5, 8], and are often only visible with two-dimensional separation techniques. We identified ten proteins on the 2D gel in two or more isoforms that were differentially regulated in cirrhotic alcoholics (Fig. (**2**); Table **2**). Other, undetected MPEFs of these proteins may be present on the gel; however, our aim was to study the proteins that were differentially regulated by liver dysfunction in alcoholics: not all proteins on the gel were subjected to MALDI-TOF MS.

Most of these proteins were found in ‘strings’ of the same molecular weight but with varying isoelectric points (pI) (Fig. (**2**)), although one protein, L-lactate dehydrogenase H subunit (LDH-B; Fig. (**2**)), was identified in two isoforms of the same pI but slightly different molecular weights. No splice variants of LDH-B are known that could produce two proteins of different sizes. This indicates that these isoforms may be post-translationally modified in a way that does not alter pI. All of these proteins are known to have phosphorylated MPEFs (Table **3**), and some are known to be methylated, acetylated, S-nitrosylated or ubiquitin-like-protein conjugated (Table **3**). The combination of these and other, yet to be discovered, modifications may be responsible for the different isoforms of these proteins observed on the 2D gel.

Many of the identified proteins are involved in basal energy metabolism (Aconitase, ACO2; ATP-synthases, ATP-syn; Glyceraldehyde-3-phosphate dehydrogenase, GAPDH; LDH-B; brain glycogen phosphorylase, PYG-B). In our study, several isoforms of ACO2, ATP-syn β, ATP-syn α  and GAPDH showed lower expression in cirrhotic alcoholics than in controls (Fig. (**2**)), indicative of an alteration in carbohydrate metabolism in brain. This metabolic change may have led to the higher expression of PYG-B in cirrhotic alcoholics (Fig. (**2**)), since this enzyme catalyses the conversion of brain glycogen to glucose. PYG-B expression may be increased as a compensatory response to reduced or impaired glycolysis. LDH-B expression was also higher in cirrhotic alcoholics (Fig. (**2**)), most likely indicating the cytotoxic effects of alcohol on the brain [9].

Ethanol administration induces 70 kDa heat shock proteins (HSP70s) expression [10, 11] as part of the protective response against alcohol-induced oxidative stress [12]. Our data suggests that only some of the MPEFs of HSP70-1 and HSP70-8 are increased in cirrhotic alcoholics; some MPEFs do not change in comparison with controls (Fig. (**2**); HSP70). Additionally, most isoforms studied showed different regulation in non-comorbid alcoholics (Fig. (**2**); HSP70; [6]). Oxidative stress is a major cause of hepatic damage in alcoholic liver disease [13], and thus an increase in certain HSP70 MPEFs in cirrhotic alcoholics implicates those particular isoforms in cerebral protection mechanisms against oxidative damage.

N-ethylmaleimide-sensitive factor (NSF) and dynamin 1 (DYN1) also demonstrated significant variation in the brain’s response to alcoholism in the presence and absence of cirrhosis. These proteins are both related to synaptic vesicle fusion and recycling; the levels of both differed significantly between non-comorbid and cirrhotic alcoholics, particularly DYN1, for which MPEFs were significantly lower in non-comorbid alcoholics than in controls [6], but not did not differ, or were slightly higher, in cirrhotic alcoholics (Fig. (**2**)). Dynamin is essential to synaptic signalling [14, 15], and the variation in DYN1 MPEF levels between cirrhotic and non-comorbid alcoholics implies that this process is differently affected in these groups.

Analysis of the MPEFs identified in this study showed significant epigenetic regulation of proteins in the superior frontal gyrus of cirrhotic alcoholics. This regulation was disease-state specific, with significant differences between MPEF regulation in non-comorbid and cirrhotic alcoholics. Further analysis of the types of post-translational modifications responsible for MPEF formation, and their regulation in different disease states, will provide insight into the mechanisms causing cerebral damage in comorbid and non-comorbid alcoholics.

## Figures and Tables

**Fig. (1):**
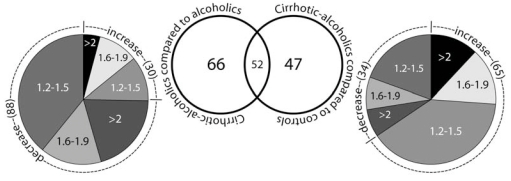
Numbers of protein expression changes between cirrhotic alcoholics and either non-cirrhotic alcoholics or controls. The pie chart shows the extent of protein that increases and decreases within three ranges: 1.2-1.5, 1.6-1.9, >2; the numbers in brackets indicate the numbers of proteins in that category. Only differences greater than 1.2 fold with a *P* value ≤ 0.05 (ANCOVA with Newman-Keuls post-hoc; values are corrected for post-mortem delay and age) are listed.

**Fig. (2):**
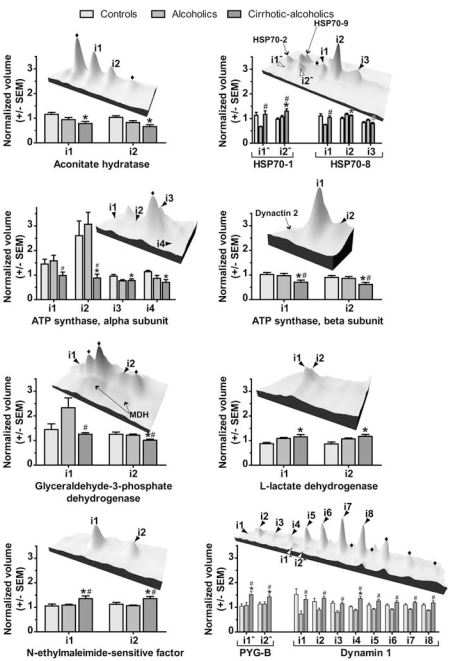
Protein expression levels in controls, non-cirrhotic alcoholics and cirrhotic alcoholics displayed in topographic and bar graph form. Graphs: average normalized volumes of each protein (derived from the average of 6 biological replicate samples per group), ± SEM. Topographic display: *, Values for cirrhotic alcoholics significantly (Newman Keuls p-value <0.05) different from controls. #, values for cirrhotic alcoholics significantly different from alcoholics. ♦, proteins positively identified as similar to the other isoforms nearby. Other protein identifications are indicated. MDH, malate dehydrogenase. PYG-B, brain variant of glycogen phosphorylase.

**Table 1:** Case Demographics

	Sex	Age (yr)	PMI (hr)	Neuropathology	Liver Pathology
**Control**	M	71	4.75	small lacunar infarct	mild microvascular steatosis
	M	75	36.5	negligible Alzheimer type changes	mild congestion
	M	67	67	old cortical infarct, L. Front cortex	acute centrilobular haemorrhage
	M	52	61.75	normal	macrovesicular steatosis
	M	89	17	normal	nd
	F	54	15.75	normal	nd
**Alcohol**	M	49	16	normal	vesicular steatosis; early fibrosis
	M	34	31	nd	mild fatty change
	M	44	22.5	mild folial atrophy, vermis	no pathology
	F	46	16.5	normal	mild fibrosis
	M	59	15	normal	moderate fatty change
	M	69	36	normal	centrilobular steatosis
**Cirrhotic alcoholics**	M	58	6	old vermal white matter lesion	hepatic cirrhosis
	M	54	24	superior vermal atrophy	cirrhosis
	M	88	14.5	normal	multinodular cirrhosis
	M	46	24	normal	cirrhosis
	M	50	24	cerebellar vermal atrophy	micronodular cirrhosis
	M	56	85	vascular defect in left Occ lobe	micronodular hepatic cirrhosis

PMI, post-mortem interval

nd, not determined.

**Table 2:** Summary of Proteins Identified in More than one Spot on the 2D Gel

	MALDI-TOF Significance Scores^a^				Alcoholics *cf.*Cirr.A^b^		Cirr.A *cf.* Controls^c^	
Isoform	MASCOT Score	Expect Score	Hits	%Cov	Diff.	P-values*	Diff.	P-values*
**ACO2; Aconitate hydratase. SwissProt Acc: Q99798**								
i1	123	1.10E-07	15	25	*1.199*	*0.184*	-1.474	0.0135
i2	125	6.90E-08	15	26	*1.247*	*0.122*	-1.563	0.0062
HSP70-1; Heat shock 70 kDa protein 1. SwissProt Acc: P08107								
i1	98	2.70E-05	11	21	-1.745	0.0021	*1.036*	*0.729*
i2	177	3.10E-13	13	36	-1.219	0.0360	1.337	0.0155
HSP70-8; Heat shock 70 kDa protein 8. SwissProt Acc: P11142								
i1	141	1.50E-09	13	38	-1.391	0.0029	*-1.061*	*0.440*
i2	162	9.70E-12	13	41	*1.052*	*0.217*	1.110	0.0267
i3	100	1.50E-05	10	29	1.174	0.0368	*-1.047*	*0.458*
ATP5A1; ATP synthase, α subunit. SwissProt Acc: P25705								
i1	71	1.60E-02	7	15	1.605	0.0488	*-1.473*	*0.058*
i2	133	7.70E-09	12	33	3.486	0.0057	-2.962	0.0100
i3	142	9.70E-10	14	32	*-1.014*	*0.872*	-1.228	0.0176
i4	80	2.10E-03	7	18	*1.226*	*0.249*	-1.611	0.0160
ATP5B; ATP synthase, β subunit. SwissProt Acc: P06576								
i1	175	6.10E-13	17	58	1.375	0.0404	-1.439	0.0478
i2	156	3.90E-11	15	59	1.391	0.0445	-1.459	0.0529
GAPDH; glyceraldehyde-3-phosphate dehydrogenase. SwissProt Acc: P04406								
i1	86	3.40E-04	8	25	1.856	0.0270	*-1.149*	*0.614*
i2	162	9.70E-12	13	39	1.205	0.0442	-1.240	0.0523
LDHB; L-lactate dehydrogenase B chain. SwissProt Acc: P07195								
i1	136	5.10E-09	14	41	*-1.095*	*0.313*	1.357	0.0188
i2	88	2.60E-04	7	22	*-1.060*	*0.499*	1.320	0.0269
NSF; N-ethylmaleimide-sensitive factor. SwissProt Acc: P46459								
i1	168	2.40E-12	18	25	-1.242	0.0368	1.287	0.0481
i2	143	7.70E-10	15	22	-1.270	0.0385	1.205	0.0445
PYGB; brain glycogen phosphorylase. SwissProt Acc: P11216								
i1	182	1.20E-13	18	27	-1.411	0.0130	1.456	0.0217
i2	64	6.40E-02	9	13	-1.259	0.0249	1.259	0.0600
Dynamin-1. SwissProt Acc: Q05193								
i1	85	6.40E-04	10	14	-1.806	0.0236	*-1.150*	*0.405*
i2	80	2.20E-03	12	16	-1.527	0.0122	*1.106*	*0.362*
i3	79	2.30E-03	13	18	-1.417	0.0043	*-1.026*	*0.767*
i4	159	2.40E-11	16	17	-1.530	0.0005	1.322	0.0022
i5	122	1.20E-07	12	21	-1.369	0.0084	*1.152*	*0.100*
i6	119	2.40E-07	12	22	-1.316	0.0067	*1.103*	*0.170*
i7	136	4.80E-09	13	22	-1.294	0.0061	*1.079*	*0.243*
i8	149	2.40E-10	15	14	-1.348	0.0172	*1.075*	*0.397*

Cirr.A, cirrhotic alcoholics.

a MALDI-TOF significance scores.

MASCOT score, a measure of the statistical significance of a match generated during the MASCOT search. Protein scores greater than the database score (NCBInr *Homo sapiens* score = 64) are significant.

Expect score, the number of times you would expect to get this score, or better, by chance

Hits, the number of experimental peptides found to match the protein in the database

% Cov, the amount that the matched peptides match the identified protein sequence.

* *P* values generated by Newman-Keuls post-hoc correction of ANCOVA values.

b A positive expression change indicates a protein that is higher in alcoholics than in cirrhotic alcoholics.

c A positive expression change indicates a protein that is higher in cirrhotic alcoholics than in controls.

Grey, italicized numbers indicate non-significant values.

**Table 3:** Known Post-Translational Modifications of Proteins Regulated in Cirrhotic Alcoholics

	Phosphorylation	Acetylation	S-nitrosylation	UBQ-like Conjugation	Methylation
**ACO2**	[16]	[17]	[16]	[18]	
**HSP70-1**	[19, 20]			[18]	
**HSP70-8**	[21, 22]				
**ATP-syn α**		[17]			
**ATP-syn β**	[21]	[17]		[18]	
**GAPDH**	[20]	[17, 23]	[24]	[18]	[25]
**LDH**	[26]			[18]	
**NSF**	[27]		[28]		
**PYG-B**	[20, 26]	[29]			
**DYN1**	[20, 30]		[31]	[32]	

UBQ, ubiquitin

ACO2, aconitate hydratase

HSP70-1 and HSP70-8, heat-shock protein 70 kDa 1 and 8

ATP-syn α  and ATP-syn β, ATP synthase α  and β subunits

GAPDH, glyceraldehyde-3-phosphate dehydrogenase

NSF, N-ethylmaleimide-sensitive factor

PYG-B, brain glycogen phosphorylase

DYN1, dynamin 1.
